# A new *VCAN/*versican splice acceptor site mutation in a French Wagner family associated with vascular and inflammatory ocular features

**Published:** 2011-06-22

**Authors:** Antoine P. Brézin, Brigitte Nedelec, Amandine Barjol, Pierre-Raphael Rothschild, Marc Delpech, Sophie Valleix

**Affiliations:** 1 Université Paris-Descartes, Centre Cochin Ambulatoire d’Ophtalmologie, Assistance Publique Hôpitaux de Paris, France; 2 Inserm, U1016, Institut Cochin, Cnrs, UMR 8104, Université Paris-Descartes, Paris, France; 3 Laboratoire de Biochimie et Génétique Moléculaire, Assistance Publique Hôpitaux de Paris, France

## Abstract

**Purpose:**

To detail the highly variable ocular phenotypes of a French family affected with an autosomal dominantly inherited vitreoretinopathy and to identify the disease gene.

**Methods:**

Sixteen family members with ten affected individuals underwent detailed ophthalmic evaluation. Genetic linkage analysis and gene screening were undertaken for genes known to be involved in degenerative and exudative vitreoretinopathies. Qualitative reverse transcriptase-PCR analysis of the versiscan (*VCAN*) transcripts was performed after mutation detection in the *VCAN* gene.

**Results:**

The first index patient of this French family was referred to us because of a chronic uveitis since infancy; this uveitis was associated with exudative retinal detachment in the context of a severe uncharacterized familial vitreoretinopathy. Genetic linkage was obtained to the *VCAN* locus, and we further identified a new pathogenic mutation at the highly conserved splice acceptor site in intron 7 of the *VCAN* gene (c.4004–2A>T), which produced aberrantly spliced *VCAN* transcripts.

**Conclusions:**

Extensive molecular investigation allowed us to classify this familial vitreoretinopathy as Wagner syndrome. This study illustrates the need to confirm clinical diagnosis by molecular genetic testing and adds new ocular phenotypes to the Wagner syndrome, such as vascular and inflammatory features.

## Introduction

Wagner syndrome (OMIM 143200) is a rare dominantly inherited vitreoretinopathy with complete penetrance, characterized by an optically “empty” vitreous with avascular vitreous strands and veils [1,2]. Typically, the disease begins in childhood, and patients with Wagner syndrome do not manifest the orofacial, skeletal and auditory features as those described in Stickler syndrome [3]. Other ocular clinical manifestations are reported in patients with Wagner syndrome, including moderate myopia, presenile cataract, ectopic fovea, inverted papilla, progressive chorioretinal degeneration with atrophy, and retinal detachments. Recently, the autosomal dominant erosive vitreoretinopathy (OMIM 143200) has been revealed to be allelic with Wagner syndrome [4-7].

The disease gene for Wagner syndrome, recently referred to as *VCAN*, codes for a chondroitin sulfate (CS) proteoglycan termed versican, which belongs to the lectican family of large aggregating chondrotin sulfate proteoglycans. Structurally, the core protein of versican is organized as a tridomain with two globular domains G1 and G3 at the N and C-terminals, respectively, and two CS attachment domains CSα and CSβ in its central area. The N- and C-terminal regions of versican contain multiple motifs that can interact with diverse extracellular matrix and cell-surface structural molecules [8,9]. Alternative splicing of exons 7 and 8 of the *VCAN* gene, coding for the glycoaminoglycan (GAG)-α and GAG-β domains respectively, generates four isoforms of versican (V0, V1, V2, and V3) with a different number of chondroitin sulfate chains and whose expressions are temporally and spatially regulated in a specific manner [10-12]. The versican V0, V1, and V3 isoforms are expressed ubiquitously and mainly in the later stages of embryonic development [13], whereas V2 is the predominant versican isoform in the mature brain [14]. The transient but elevated levels of versican expression in various embryonic tissues suggest a central role of this molecule in tissue morphogenesis, while its decreased expression after tissue maturation indicates that versican’s function is implicated in the control of tissue homeostasis [15,16].

There is considerable evidence that differential expression of the lectican family of chondrotin sulfate proteoglycans may be important factors for modulating cell adhesion as well as axonal growth and guidance during neural development [17]. In the eye, versican’s expression is found in the vitreous, the retina, and also in the ciliary body and the trabecular meshwork, but its exact role in these different ocular tissues remains largely unknown. In the vitreous, versican is incorporated into the ECM and forms large aggregates that support vitreous integrity by maintaining the spatial organization of hyaluronan and collagen fibers. Versican expression, especially the V0 isoform, which has the highest number of chondroitin sulfate attachment sites, is found in the nerve fiber layer, and the inner plexiform layer during chicken retinal development, while it is distributed in the inter-photoreceptor matrix in adult chicken retina [18].

To date, only nine Wagner families have been reported with the identification of the molecular defect in the *VCAN* gene. A total of six different causative mutations, all affecting either the conserved acceptor splice site of intron 7 or the donor splice site of intron 8 of the *VCAN* gene, have been identified [7,19-22]. It has been demonstrated that these splice site mutations responsible for Wagner syndrome lead to skipping of exon 8, yielding an imbalanced quantitative ratio of versican transcripts with an increased amount of V2 and V3 isoforms and haploinsufficiency of V0 and V1 isoforms [7,19,21]. Therefore, the pathogenic mechanism of Wagner syndrome is thought to result in a reduction of chondroitin sulfate side chains, which affect interactions of versican, with other extracellular components responsible for accelerated pathologic liquefaction of the vitreous gel. To better understand the role of versican in the physiologic and pathological processes in which it is suspected to be involved, more Wagner families need to be characterized.

We report a four-generation French family presenting a severe vitreoretinal disorder inherited as an autosomal dominant trait without evident systemic clinical features. The first index patient was referred to us because of a chronic uveitis associated with exudative retinal detachment in the context of a severe uncharacterized familial vitreoretinopathy. The clinical diagnosis of the disease presented by this family was controversial, but genetic linkage was obtained to the gene for Wagner syndrome. Subsequently, we identified a novel acceptor splice site mutation in intron 7 of the *VCAN* gene (c.4004–2A>T) that produces aberrantly spliced *VCAN* transcripts identical to those found in the Japanese Wagner family with the (c.4004–2A>G) *VCAN* splice site mutation [21]. Here, we also detail unusual ocular findings, which extend the phenotypic characteristics of Wagner syndrome.

## Methods

### Patients

The study followed the tenets of the Declaration of Helsinki and was approved by the ethics review committee of the Institutional Review Board of Cochin Hospital (Paris, France). Informed consents were obtained from all participating patients before donation of blood samples. Complete ophthalmologic examinations were performed in affected and unaffected family members. Kinetic perimetry was tested with the Goldmann perimeter (Haag-Streit AG, Bern, Switzerland), and fundus photography was performed when pupil dilatation was sufficient (Canon fundus camera with Megaplus model 1.4 digital imaging system, Tokyo, Japan). Optical coherence tomography imaging was obtained with the Stratus OCT (Carl Zeiss, San Leandro, CA) and full-field electroretinography was performed on selected affected patients with the MonElec2 Monitor Model (Metrovision, Pérenchies, France). Anterior chamber flare was quantified with the Kowa FM 500 flare meter (Kowa Company Ltd., Tokyo, Japan).

### Linkage analysis

Genotyping was performed using fluorescently tagged microsatellite markers, which were previously reported or were selected from the Marshfield human genetic linkage map. These markers are closed to the genes for Collagen, type II, α-1 (*COL2A1*; D12S1713, D12S1620), Frizzled, *Drosophila*, homolog of, 4 (*FZD4*; D11S527, D11S896), low density lipoprotein receptor-related protein 5 (*LRP5*; D11S4076, D11S4113), and versican (*VCAN*; D5S626, D5S641, D5S107, D5S2103). Linkage analysis was performed under the assumption of a dominant model with 100% penetrance and 0.0001 frequency of the disease allele. Two-point linkage analyses were performed using the MLINK program from the Linkage package, version 5.1.

### Mutation screening

DNAs were extracted from peripheral blood samples, using the QIAamp DNA mini kit (Qiagen, Valencia, CA). Exons and intron–exon junctions for the *FZD4* (two exons, four amplicons), *LRPR5* (23 exons, 23 amplicons), norrin (*NDP*; three exons, two amplicons), tetraspanin 12 (*TSPAN12*; eight exons, seven amplicons) genes and exons 6, 7, 8, and 9 of the *VCAN* gene were amplified by PCR. PCR primers for *VCAN*, *FZD4,* and *NDP* exons were developed using the Primer 3 software, and all these sequences are listed in Table 1, whereas PCR primers for *TSPAN12* and *LRP5* exons were designed as described previously [23,24]. Sequencing reactions were performed in an automatic genetic analyzer (ABI PRISM 3100 genetic analyzer; Applied Biosystems, Foster City, CA), using the Big Dye terminator cycle sequencing kit (DNA sequencing kit; Applied Biosystems).

**Table 1:** Primer sequences for PCR amplification

**Exons**	**Primer name**	**Primer sequence (5′-3′)**	**Product size (bp)**
***VCAN***			
6	VCAN_6F	tgctcctccaattccttcc	482
	VCAN_6R	acatggaatccactccaacg	
7	VCAN_7–1F	agcacttaacaaacactgggc	618
	VCAN_7–1R	ttccaggatcattcttgcag	
7	VCAN_7–6F	caggatttacatcatctttgagtcc	666
	VCAN_7–6R	tcttctaatagcagcatttggtatg	
8	VCAN_8–1F	gaccagccttgctatcaatttc	605
	VCAN_8–1R	ttcctcatggaatgggactg	
8	VCAN_8–11F	aatggaagcttctcccacag	600
	VCAN_8–11R	acatataaacgttagcaccaagg	
9	VCAN_9F	cacgcaaacattagagacgag	243
	VCAN_9R	tcgacaacataaattctcccc	
***FZD4***			
1	FZD4_1–2F	gtcgccgcatcacactc	516
	FZD4_1–2R	ctgtctccttcgggctagg	
2	FZD4_2–1F	tggtcaaacttccaagtcattg	615
	FZD4_2–1R	gatatcctttcccggcctac	
2	FZD4_2–2F	tgaccttcctgatcgattcttc	601
	FZD4_2–2R	accccaatcttgaccatcag	
2	FZD4_2–3F	ggaactttgttcattgctgc	630
	FZD4_2–3R	aactggcttttccattttgg	
***NDP***			
2	NDP_2F	ggatcctaggaggtgaagcc	339
	NDP_2R	ttctccatcccctgacaaag	
3	NDP_3F	caacgagtgtgagggtctttc	397
	NDP_3R	ttcttgccagtctttccctg	

### Qualitative RT–PCR analysis

Total RNA was extracted from cultured lymphoblastoid cells from two affected family member’s (V.5 and IV.8) and one control unaffected subject (V.4), with a commercial kit (RNA Plus; Q.BIO gene, Montreal, Canada). cDNA was synthesized from 1 µg total RNA using random hexamers and M-MLV reverse transcriptase (RT, Superscript III; Invitrogen, Carlsbad, CA). RT–PCR was performed using different sets of primers previously designed for specific amplification of each of the four versican isoforms [21].

## Results

### Ocular phenotypes

**Figure 1:**
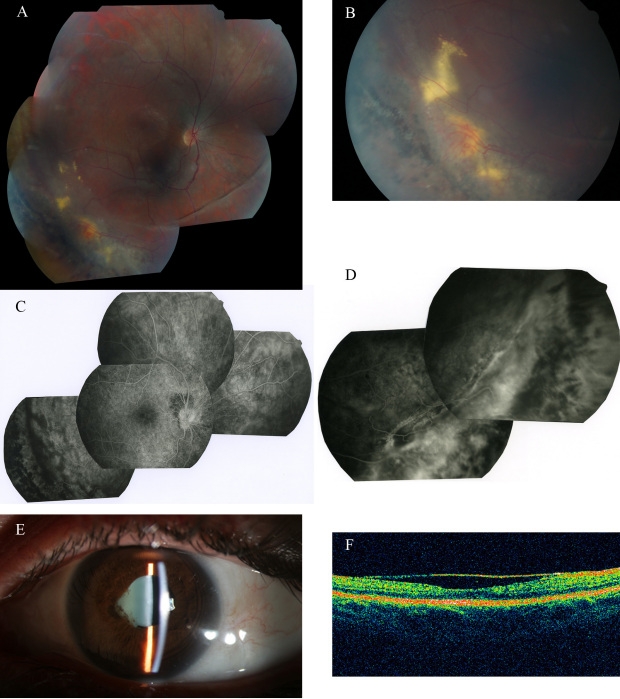
Patient V.5 (the first index case of the family). **A**: Mosaic multifield fundus photograph of the patient’s right eye, showing an avascular vitreous veil, approximately 2–3 disc diameters anterior to the inferior temporal retinal vein, with patches of chorioretinal atrophy, retinal pigmentary, and exudative lesions. **B**: Exudative yellow lesions with telangiectatic Coats-like neovascularization are seen in the retinal periphery. **C**: Fluorescein angiogram of the right eye of patient V.5 showing the superior pole and superior nasal and inferior temporal periphery. A mildly titled disc is seen. The retinal vasculature appears normal at the posterior pole and in the superior nasal periphery, and pigment clumping is present in the inferior temporal field. **D**: Fluorescein angiogram of the right eye of patient V.5 showing pigment clumping parallel to the ora in the inferior nasal periphery. Peripheral vascular leakage is seen as diffuse areas of hyperfluorescence. **E**: Slit-lamp photograph of the left eye of patient V.5 showing iris–lens synechiae from the 7:00 to the 10:00 position. **F**: OCT imaging of the right eye of patient V.5 showing a dense posterior hyaloid forming a bridge over the foveal pit.

Patient V.5, our index case, was a 29-year-old female patient who had been referred to our ophthalmologic center for repetitive anterior uveitis with iris–lens synechiae (Figure 1E). She was moderately myopic and her intraocular pressure (IOP) was slightly increased with 18 mmHg in her right eye (OD) and 22 mmHg in her left eye (OS). Laser flare meter measures were four to five times above normal values: OD 38.5 and OS 50.3 photons/ms. Fine keratic precipitates were observed in both eyes (OU), and there were 0.5+ cells in the anterior chamber of the right eye and 2+ cells in the left eye. The patient had a history since the age of 12 of recurrent alternating attacks of uveitis. These attacks were successfully treated by topical dexamethasone drops within 7–15 days after the onset of attacks. Additionally, this patient was followed since early childhood for bilateral vitreoretinal lesions with peripheral exudative retinal detachments (RDs). Fluorescein angiography, performed at age 22, did not show evident interruption of the peripheral retinal vasculature but revealed numerous peripheral areas of exudation, which appeared inhomogeneously hyperfluorescent (Figure 1C,D). At that time, the diagnosis of her retinal disease was classified as familial exudative vitreoretinopathy and she had been treated by bilateral cryotherapy at age 7. In our ophthalmologic center, her fundus examination showed an optically empty vitreous with the presence of an annular vitreous veil parallel to the ora in both eyes (Figure 1A). Peripheral exudates associated with areas of pigmentation and neovascularization were predominantly present in the temporal sector of the retina of both eyes (Figure 1B). Scars of cryotherapy and laser were visible in the inferior and superior temporal peripheral retinas. An annular reflex was seen at the periphery of the macular area, and OCT imaging showed a densification of the posterior hyaloid, forming a bridge over the foveal pit (Figure 1F). Goldmann manual perimetry showed normal peripheral boundaries in the temporal, superior, and inferior quadrants, but the nasal position of the V1e isopter, which was 55° for the right eye, was mildly constricted to 42° for the left eye. Electroretinography revealed decreased scotopic and photopic a and b waves, with predominant left eye involvement (data not shown). An extensive workup to exclude other causes of uveitis was negative. In particular, the patient did not carry the HLA B27 allele, her chest CT-scan and serum angio-convertin enzyme levels were normal, and numerous serologies for infectious causes of uveitis were negative.

**Figure 2:**
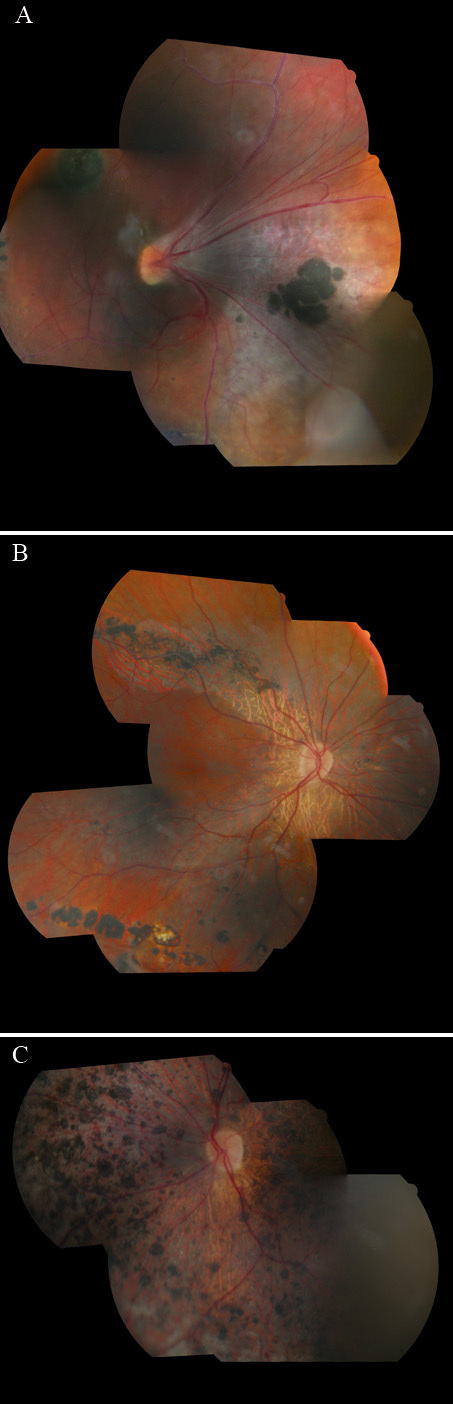
Mosaic multifield fundus photographs of different family members. **A**: Fundus photograph of patient V.2 showing a nasal dragging of the retinal vessels and fovea with white perivascular sheathing. Clumps of pigment are visible in the nasal retina. No avascular vitreous membrane was seen. **B**: Fundus photograph of the right eye of patient V.6 showing a normal retinal vasculature with atrophy and pigment clumping along and anterior to the temporal arcades. No avascular vitreous membrane was observed. **C**: Fundus photograph of the left eye of patient V.6 showing diffuse pigmentation with chorioretinal atrophy.

Patient V.2, a 31-year-old female first cousin of the index case, had a BCVA of 0.4 in her right pseudophakic eye and 0.25 in her left eye. Because of her family medical history, her first ophthalmologic examination was performed at the age of 1 year and showed vitreoretinal exudative lesions; the disease was classified as familial exudative vitreoretinopathy. Bilateral peripheral cryotherapy was performed at the age of 9 years and was followed by an RD in her left eye, which was treated by a scleral buckling procedure. An RD of the left eye was treated by vitrectomy and cryotherapy at the age of 13 years. Cataract surgery by phacoemulsification with placement of a posterior chamber IOL was performed at the age of 29 years in her right eye. At the time of our examination, her IOP was 12 mmHg OD and 16 mmHg OS. Laser flare photometry was minimally increased to 14 photons/ms OD and more significantly to 39 photons/ms OS. Fundus examination showed a bilateral nasal stretching of retinal vessels with ectopic foveae and nasal pigment clumping (Figure 2A). The vitreous appeared clear without veils or strands. OCT imaging showed a similar profile as our index case, with a densification of the posterior hyaloid forming a bridge over the foveal pit.

Patient V.6, a 36-year-old female first cousin of the index case, had a BCVA of 0.6 OD and no light perception in her left eye. She had a history of RD in her left eye, which was diagnosed at age 6 and which was unsuccessfully operated on. Although she had no light perception in that eye, a white cataract was removed at the age of 33 years for esthetic purposes, without placement of an IOL. Cryotherapy in her right eye was performed at the age of 8 and 12 years, and this was later followed by laser photocoagulation of the retinal periphery. At the time of our examination, laser flare photometry was minimally increased to 12.5 photons/ms OD and more significantly to 32.3 photons/ms in her left aphakic eye. Examination of the fundus showed a clear vitreous without strands or veils, and vessels did not appear stretched. Optic disc pallor was observed in the left eye only. In the right fundus, retinal atrophy was seen in the peripapillary area and along the temporal vascular arcades, and this was associated with pigment clumping (Figure 2B). In the left eye, the chorioretinal atrophy was more diffuse and the retina appeared grayish and speckled with pigment clumping, giving an aspect of pseudo-retinitis pigmentosa. OCT imaging showed a normal macular profile OD and a thinned atrophic retina OS.

The medical history and ocular phenotypes of affected family members of the fourth generation are summarized in Table 2. At the time of examination in our center, all these patients were blind and laser flare meter measurements were found to be increased. All had manifested RDs at an early age, with variable anterior segment manifestations, including moderate myopia, glaucoma, cataracts, and ectopia lentis.

**Table 2:** Summary of clinical characteristics of patients with the intron 7 splice acceptor mutation of the CSPG2 gene (c.4004–2A>T)

**Patient**	**Age**	**Visual acuity**	**Main clinical findings**
II.1 and III.2			Reported to be blind at an early age, secondary to RDs
III.4*	82	NLP / NLP	OD: RD, Prosthesis OS: Aphakic, glaucoma, retinis pigmentosa-like fundus
IV.2	64	NLP / LP in 1 quadrant	OD: Aphakic, phtisis bulba, RD at age 33, retinis pigmentosa-like fundus, vitreous veils OS: RD at age 11, Prosthesis
IV.4	63	NLP / NLP	OD: RD at age 3, Prosthesis OS: Aphakic, phtisis bulba
IV.6	60	0.2 / NLP	OD:Pseudophakic OS: Cataract, glaucoma (2 trabeculectomies), prosthesis Chorioretinal atrophy, pigment clumping, vitreous strands
IV.7	57	0.5 / NLP	OD: Aphakic OS: RD at age 7, phtisis bulba, diffuse band keratopathy, nasally stretched retinal vessels, temporal pigmented lesions
IV.8	46	HM / 0.5	OU: Pseudophakic Chorioretinal atrophy, diffuse pigment clumping
IV.9	46	NLP / 0.6	OD: RD at age 7, prosthesis OS: Pseudophakic peripheral cryotherapy and laser scars
V.2	31	0.4 / 0.25	OD: Pseudophakic, RD at age 13 OS: vitreoretinal exudative manifestations, RD at age 9 OU: Nasally stretched retinal vessels, pigment clumping, ectopic faveae, no vitreous strands
V.5	29	0.6/0.4	OU: Repetitive anterior uveitis with synechiae Retinal vascular abnormalities, peripheral exudates in temporal retina, pigment clumping, vitreous fold parallel to the ora
V.6	36	0.6/NLP	OS: Aphakic, RD at age 6, chorioretinal atrophy, pseudo-retinis pigmentosa OD: Pigment clumping, retinal atrophy in the peripapillary area and along the temporal vascular arcades, no vitreous strands

* Not examined in our center. Abbreviations: LP represents light perception ; NLP represents no light perception, HM represents vision limited to the perception of hand motion, RD represents retinal detachment.

### Linkage analysis

**Figure 3:**
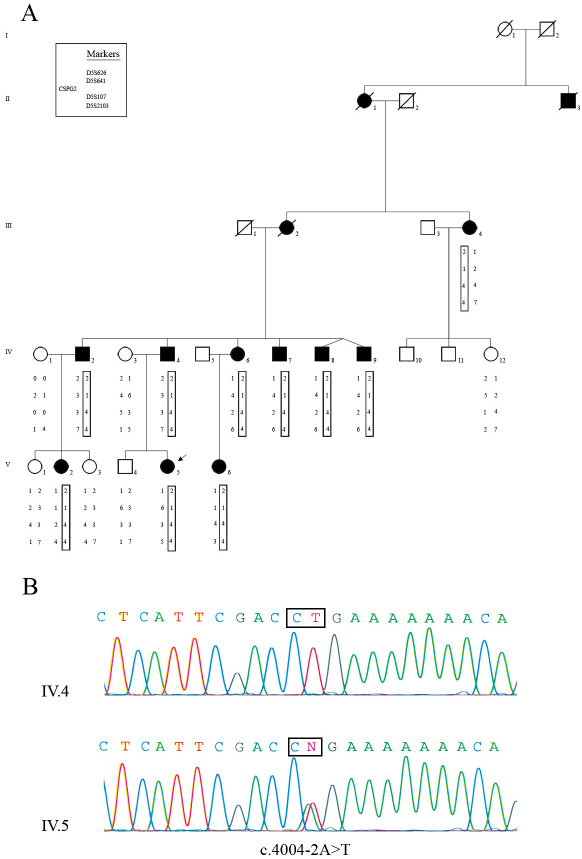
Pedigree of the Wagner family, linkage and mutation analysis of the versican (*VCAN*) gene. **A**: The pedigree of the family studied consisted in ten affected and six unaffected participating individuals, providing strong evidence of autosomal dominant mode of inheritance. Filled symbols represent affected individuals, whereas clear symbols refer to unaffected individuals. The arrow indicates the first proband examined in our ophthalmologic center. Segregation analysis of microsatellite markers encompassing the WGN1 locus on chromosome 5q14.3. The disease-associated haplotype is boxed and is shared between all affected members. The genetic markers and the corresponding allele are listed in map order. **B**: Partial sequence chromatograms of intron 7–exon 8 boundary of the *VCAN* gene from one affected individual (V.5) and one normal control subject (V.4) showing a heterozygous adenine to thymine transversion at the second base of the 3′ acceptor splice site of intron 7 (c.4004–2A>T).

As a first step toward identifying the causative gene in this four-generation French family, we performed linkage analyses. Sixteen family members, including ten affected individuals and six unaffected individuals, were genotyped, and significant evidence of linkage was shown with markers D5S641 (Z=3.36, ø=0) and D5S2103 (Z=2.89, ø=0), indicating a critical disease region for the WGN1 locus on 5q13-q14 (Figure 3A).

### DNA sequence analysis

Direct sequencing of the *VCAN* gene from the index patient (V.5) DNA sample revealed a novel heterozygous A>T transversion at the second base of the 3′-acceptor splice site of intron 7 (c.4004 −2A>T; Figure 3B). This base pair substitution was present in the ten affected patients of this family and absent in six unaffected family members, confirming that it co-segregated with the disease status. Furthermore, this splice site mutation was not detected in 100 unrelated French control subjects, supporting that this sequence variation is pathogenic and is not a rare polymorphism. This mutation occurred in a highly conserved 3′-acceptor splice site of intron 7 and is theoretically expected to destroy this canonical splice site sequence, causing exon 8 skipping and/or activation of a downstream cryptic acceptor splice site within exon 8 of the mutant allele. Because of exudative vascular features in some patients of this family, we also undertook mutation screening in *FZD4*, *LRP5*, *NDP,* and *TSPAN12* genes, but no pathological mutations were identified.

### *VCAN* transcript analysis

**Figure 4:**
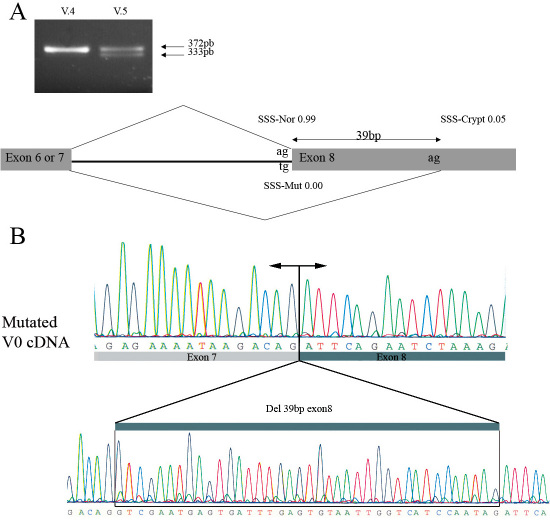
Versican (*VCAN*) transcript analysis. **A**: Ethidium bromide-stained agarose gel of reverse transcription- polymerase chain reaction (RT–PCR) products obtained with primer pairs 7F/8R for amplification of the versican V0 mRNA from cultured lymphoblasts of one affected individual (V.5) and one control unaffected subject (V.4). The results indicate that both a wild-type (band of 372 bp) and a 39-nt deleted (band of 333 bp) cDNA fragment of the V0 transcript are present in the affected patient harboring the intron 7 splice acceptor mutation of the *VCAN* gene (c.4004–2A>T). The nucleotide change at the 3′ acceptor splice site of intron 7 of the *VCAN* gene was analyzed using the NNSplice program for prediction in alteration of splicing junctions, and the splice-site scores (SSSs) for a normal site (SSS-nor), a mutated site (SSS-mut), and a cryptic site (SSS-crypt) were calculated [28]. **B**: Partial sequence chromatogram of this aberrant V0 transcript lacking the first 39 bp of *VCAN* exon 8.

The effects of this novel *VCAN* intron 7 splice site mutation were assessed by RT–PCR analysis from immortalized lymphoblastoid cell total RNAs generated from two affected individuals (V.5 and IV.8) and one control subject (V.4). Results of RT–PCR indicated that the c.4004–2A>T mutation activates a cryptic acceptor splice site within exon 8, located 39 bp downstream from the authentic 3′ splice acceptor site. Indeed, electrophoresis in 3% agarose Nusieve gel of RT–PCR products obtained with primer pairs 7F/8R revealed a band of 372 bp and a slightly shorter fragment of 333 bp in RNA sample from the index patient, but only one fragment of 372 bp in the control RNA sample (Figure 4A). Direct sequencing of this shortened abnormal fragment indicated the loss of the first 39 base pairs of exon 8 from the mutant allele; this loss was expected to lead to an in-frame deletion of 13 amino acid residues from the beginning of the GAG-β domain of the mature versican (Figure 4B). Additionally, RT–PCR analysis with primer pairs 7F/9R revealed a band of 286 bp in the RNA sample from the index patient, which was not detected in the RNA control sample, indicating an aberrant expression of a versican mRNA lacking the entire exon 8 (data not shown).

## Discussion

We report the first French family with ocular features of Wagner syndrome segregating with a adenine to thymine transversion at the highly conserved splice acceptor site in intron 7 of the *VCAN* gene (c.4004–2A>T). This new single base pair substitution affects the same nucleotide of the intron 7 splice acceptor site that had been substituted into a guanine (c.4004–2A>G) in a previously reported Japanese Wagner family [21]. In the present study, we could confirm that our new nucleotide substitution produces aberrantly spliced *VCAN* transcripts identical to those found in the Japanese Wagner family [21].

This large single kindred demonstrated a range of highly variable phenotypes, from exudative vascular abnormalities and diffuse retinal atrophy with pigment clumping to nasally deviated retinal vessels with ectopic foveae, leading to different initial diagnoses of the disease until characterization of the molecular defect in the *VCAN* gene was achieved. Exudative vascular features are unusual phenotypic findings in Wagner syndrome, and the exudative component was significant enough that the initial diagnosis of autosomal dominant exudative vitreoretinopathy (FEVR) in our index case was highly suspected. Indeed, this patient, who was born at term with normal birthweight and who did not received oxygen therapy, showed peripheral retinal vascular abnormalities with severe retinal exudates leading to exudative retinal detachment, telangiectatic neovascularization, peripheral chorioretinal pigmentation, straightening of the retinal blood vessels, and situs inversus similar to a FEVR-like phenotype. To exclude genetic heterogeneity in the kindred studied here or a synergistic effect of an additional mutation in a gene implicated in FEVR, we performed mutation screening of *FZD4*, *LRP5*, *NDP*, and *TSPAN12* genes, which are known to be responsible for FEVR. We finally demonstrated that these heterogeneous clinical presentations exhibited by this single French kindred are linked to the same molecular defect in the *VCAN* gene. The visual impairment of the disease was highly significant in our pedigree since, among ten affected family members, three are totally blind and five other patients have completely lost vision in one eye. RDs were particularly frequent and occurred at an early onset in the family studied here, and the RDs also had a poor surgical prognosis. Therefore, RD in Wagner syndrome appears to be a more common feature and one that occurs earlier than previously suggested [22]. Our first index patient (V.5) had an exudative RD at the age of 30 years, with vascular abnormalities resembling Coat’s telangiectasies in her temporal peripheral retinas. This is the second known case of such vascular lesions that have previously been described in the original Wagner family [1]. Our index patient also presented with recurrent episodes of bilateral anterior uveitis with increased laser flare meter measurements; all these signs are indicative of a breakdown of the blood–retinal barrier. Although inflammation is not typical of Wagner disease, episodes of bilateral anterior uveitis in two family members affected with Wagner syndrome have been described [20]. Therefore, the family studied here represents the second case of uveitis associated with Wagner disease. However, due to the low number of Wagner families that have been molecularly recognized, it remains difficult to determine whether uveitis is part of the disease or rather uveitis occurred coincidentally in the two unrelated families. Interestingly, different recent studies support a role for versican in controlling inflammation and angiogenesis. Indeed, versican is known to be an abundant proteoglycan in the blood vessel wall, and it has also been demonstrated that the C-terminal G3 domain of versican forms a ternary complex with fibronectin and VEGF, thereby promoting angiogenesis [15,25,26]. Also of interest, a recent study has documented that versican can elicit the production of pro-inflammatory cytokines via its interaction with toll-like receptor 2 [27]. Moreover, the CS chains attached to the GAG-α and GAG-β domains of versican are involved in the binding of a series of cytokines, suggesting that an appropriate balance of each splice isoform of versican may be important for regulating inflammatory responses [9].

In conclusion, molecular investigation of this French family allowed us to classify this severe uncharacterized familial vitreoretinopathy as Wagner syndrome. This study illustrates the necessity to confirm clinical diagnosis by molecular genetic testing and adds new ocular phenotypes to the Wagner syndrome, such as vascular and inflammatory features. Therefore, phenotype–genotype studies of additional Wagner patients are necessary to establish whether such vascular and inflammatory features are consistent observations in Wagner disease.
